# Exercise Ameliorates Endocrine Pancreas Damage Induced by Chronic Cola Drinking in Rats

**DOI:** 10.1371/journal.pone.0155630

**Published:** 2016-05-18

**Authors:** Matilde Otero-Losada, Julián González, Angélica Müller, Graciela Ottaviano, Gabriel Cao, Francisco Azzato, Giuseppe Ambrosio, José Milei

**Affiliations:** 1 Instituto de Investigaciones Cardiológicas, Universidad de Buenos Aires, Consejo Nacional de Investigaciones Científicas y Técnicas, ININCA.UBA.CONICET, Buenos Aires, Argentina; 2 Università di Perugia, Cardiologia e Fisiopatologia Cardiovascolare, Perugia, Italy; University of Szeged, HUNGARY

## Abstract

**Purpose:**

This study evaluates whether the daily practice of an exercise routine might protect from endocrine pancreas damage in cola drinking rats.

**Methods:**

Forty-eight Wistar rats were randomly assigned to 4 groups depending on a) beverage consumption *ad libitum*, water (W) or cola beverage (C), and b) physical activity, sedentary (S) or treadmill running (R). Accordingly, 4 groups were studied: WS (water sedentary), WR (water runner), CS (cola sedentary) and CR (cola runner). Body weight, nutritional data, plasma levels of glucose, creatinine, total cholesterol and cholesterol fractions, and triglycerides (enzymocolorimetry), and systolic blood pressure (plethysmography) were measured. After 6 months, euthanasia was performed (overdose sodium thiopental). Pancreatic tissue was immediately excised and conventionally processed for morphometrical and immunohistochemical determinations.

**Results:**

The effects of running and chronic cola drinking on pancreas morphology showed interaction (p<0.001) rather than simple summation. Cola drinking (CS vs WS) reduced median pancreatic islet area (-30%, 1.8 10^4^ μm^2^ vs 2.58 10^4^ μm^2^, p<0.0001) and median β-cell mass (-43%, 3.81 mg vs 6.73 mg, p<0.0001), and increased median α/β ratio (+49%, 0.64 vs 0.43, p< 0.001). In water drinking rats (WR vs WS), running reduced median α-cell mass (-48%, 1.48 mg vs 2.82 mg, p<0.001) and α/β ratio (-56%, 0.19 vs 0.43, p<0.0001). Differently, in cola drinking rats (CR vs CS), running partially restored median islet area (+15%, 2.06 10^4^ μm^2^ vs 1.79 10^4^ μm^2^, p<0.05), increased median β-cell mass (+47%, 5.59 mg vs 3.81 mg, p <0.0001) and reduced median α/β ratio (-6%, 0.60 vs 0.64, p<0.05).

**Conclusion:**

This study is likely the first reporting experimental evidence of the beneficial effect of exercise on pancreatic morphology in cola-drinking rats. Presently, the increase of nearly 50% in β cells mass by running in cola drinking rats is by far the most relevant finding. Moderate running, advisably indicated in cola consumers and patients at risk of diabetes, finds here experimental support.

## Introduction

Metabolic syndrome (MetS) is a cluster of conditions: hypertension, hyperglycemia, overweight and dyslipidemia that pose a high risk of cardiovascular disease, stroke and diabetes. Occidental urban societies have high prevalence of cardiovascular disease, diabetes and obesity [1–4]. The striking increase in the prevalence of MetS and diabetes, diabetes risk and cardiovascular complications in the last decades has been related to regular consumption of sugar-sweetened soft beverages as observed by meta-analyses and epidemiological studies [2,3,5,6]. Consumption of sugar-sweetened sodas might influence MetS development through accelerated cell aging [7].

The number of diabetic patients with obesity has increased due to life-style changes which favor sedentarism and unhealthy nutritional habits [8,9]. Fortunately, by now, an increasing number of people are integrating a regular exercise practice into everyday life, as a way to compensate for bad nutritional habits and improve health [10]. Hopefully, regular consumers of large volumes of soft drink and poor quality food are becoming aware of the benefits from practising a daily routine of aerobic moderate exercise.

In diabetic patients, aerobic exercise has been found not only to reduce hypertriglyceridemia and hyperglycemia but to contribute to weight-loss as well [11,12]. In rats, the regular practice of exercise contributed to keep glucose homeostasis, to promote β-cell function [13,14], and to normalize lipid metabolism [15,16]. Weight loss is actually an essential factor to improve health status in diabetic patients [17]. The Newcastle University and the University of Glasgow are by now carrying on a conjoint research, the DiRECT (DIabetes REmission Clinical Trial) study, comparing the long-term effects of a new weight management approach to the best diabetes care currently available, and results will be available not until October 2018. Previous confirmatory reports show that type 2 diabetes is not inevitably progressive and life-long and that, in many diabetics with a history of illness of up 10 years, major weight loss returns insulin secretion to normal [18]. Normoglycemia can be achieved in long-duration (>8 yrs) type 2 diabetes, but a greater degree of weight loss (>25kg) is required than for short-duration (<4 yrs) diabetes [19].

So far, no experimental research has been conducted in order to evaluate the effects of combining an exercise routine with a nutritional factor on endocrine pancreas. Actually, even the individual effects of exercise on endocrine pancreas have been scarcely addressed not to mention that alpha cells have not been evaluated either.

Nearly for ten years, we have been studying the variety of physiological and morphological alterations found after chronic ingestion of cola beverages in both genetically modified mice and normal rats. We found that chronic cola drinking accelerated aortic atherosclerosis and enlarged atherosclerotic lesions in atherosclerotic mice [20,21]. In healthy rats, chronic cola drinking induced biochemical and echocardiographic changes, namely, dyslipidemia, hyperglycemia, cardiac remodelling with left ventricle hypertrophy and increased cardiac output [22,23].

Recently we reported that 6 months of cola-drinking induce MetS-like features: hyperglycemia, hypertriglyceridemia, mild overweight and insulin resistance, and affects endocrine pancreas morphology in rats [22–24].

Hence, consistently with our line of research on experimental metabolic syndrome mimicking life-style conditions concerning with a human nutritional habit as chronic cola-drinking, we conceived a model combining the systematic practice of an aerobic exercise with cola-drinking, a combination frequently observed these days in human life-style.

This study evaluates whether an aerobic exercise program may help to ameliorate metabolic disorder and preserve normal morphology in pancreatic islets of cola-drinking rats.

## Material and Methods

The experiment was conducted in accordance with the recommendations of the Weatherall report, "The use of non-human primates in research." The committee of Ethics in Animal Research of the Instituto de Investigaciones Cardiológicas (ININCA) and the Institutional Animal Care and Use Committee (IACUC) of the Faculty of Medicine of the University of Buenos Aires, namely the CICUAL (Institutional Committee for the Care and Use of Laboratory Animals) approved the study.

In regard to our previous publications [24,25], different rat batches from the same breeders were used in the respective experiments at, of course, not only different seasons but different years as well. The experimental design in the present study is analogous to that reported recently in this journal [25] though different sets of animals were evaluated for respectively different purposes according to the respective topics of the study. Rats whose results are reported presently, were exclusively evaluated in the current experiment, they did not participate in any other one and there is no overlapping between rats in current and previous studies of ours. Besides, In accord with the Publication Criteria of PLOS ONE, we declare there are no other submitted/accepted/published publications related to the topic of this article.

Forty-eight adult male Wistar rats were randomly assigned to two groups depending on beverage consumption “ad libitum”: water (W) or cola (C, Coca-Cola^™^, Argentina). In turn, each group was split into two subgroups depending on physical activity: S (sedentary) or R (runner). Accordingly, 4 groups were studied (n = 12 each): WS, WR, CS and CR for 6 months. Animal care followed the ‘Guide for the Care and Use of Laboratory Animals’ (NIH publication n°85–53, revised 1998).

Rats were weighed weekly. Food and drink consumption were assessed twice a week. At 0 and 6 months, systolic blood pressure (SBP) was measured by tail cuff plethysmography, and biochemical determinations were performed in blood collected from the tail vein after 4-hour fasting. Commercially available kits for enzymatic colorimetric assays (Sigma-Aldrich, USA) were used to measure concentration of: glucose (hexokinase/glucose-6-phosphate dehydrogenase reaction), creatinine (colorimetric, alkaline picrate method), total cholesterol (enzymatic colorimetric test), HDL-c (selective accelerating detergent), LDL-c (homogeneous enzymatic colorimetric assay) and triglycerides (glycerophosphate oxidase). The atherogenic index in plasma was calculated as AIP = log (TG/HDL-c) [26].

The aerobic exercise program consisted in treadmill running on a 10° inclined plane for 30 minutes daily, 5 times per week, at a maximal speed of 20 m/minute (treadmill Columbus^™^) [27].

After 6 months, rats were euthanized by subtotal exsanguination (bleeding of the coccygeal artery after tail warming) under anesthesia (sodium thiopental 40 mg/kg, i.p.). At autopsy, pancreas were removed, weighed, fixed in phosphate-buffered 10% formalin solution and processed for histology.

Eight 3 μm sections of tissue blocks were stained with hematoxylin-eosin (H-E). For immunohistochemistry, the traditional streptavidin-biotin-peroxidase complex technique was used. A blocking solution (4% dry skim milk and 3% bovine serum albumin, BSA, Sigma A7030) was used to block non-specific protein binding by incubation for 30 min at 37°C and 90 min at room temperature, in phosphate-buffered saline solution (PBS) at pH = 7.4. Overnight incubation at 4°C with primary monoclonal mouse antibodies against insulin (dilution 1/3000 in blocking solution) and glucagon (dilution 1/30000 in blocking solution) (Sigma-Aldrich) allowed labeling of β- and α-cells, followed by incubation with biotinilated goat anti mouse secondary antibody (diluted 1/500 in PBS) 2h at room temperature. Finally, streptavidin (1/1000 in PBS) was added for 1h at room temperature, followed by addition of chromogen DAB 3-5min. Control sections were incubated with non-immune normal mouse serum.

Islet cross-sectional area was estimated counting the number of points hitting an islet (point-counting method) in ≥ 30 islets/pancreas. An orthogonal grid with 300 test points projected onto the fields of view representing an area of 6.7 10^4^ μm^2^ at 40 X objective lens. Alpha- and β-cell fractional area was calculated as immunopositive-to-total islet area ratio for glucagon and insulin respectively and it was expressed as percentage. Alpha- and β-cell mass was estimated as the product of the relative cross-sectional area of β cells per total tissue and the weight of the pancreas [28]. Images were analyzed using Image-Pro Plus 6.0 (Media Cybernetics, Silver Spring, Maryland, USA).

For variation of a given factor which is expressed in %, e.g. from 2% to 3%, stating that there is “a 1% increase” is ambiguous for it might be interpreted in two ways: a). the difference is 1% unit b). the change represents 1% of the initial or reference value. In the above example: the absolute change is 1% unit (3–2) while the relative change is 50% (= 100 x [3–2]/2). In our paper, fractional area changes are expressed in both ways, e.g.: the effect of cola drinking on beta-cell fractional area which varies from 58.71 to 44.47, that is expressed as a 24% change (14.24% units). Relative change = 1-[100 x (44.47–58.71)/58.71] = 24%, absolute change = 44.47–58.71 = 14.24% units [29].

### Statistical analysis

Biochemical values (parametric variables) were expressed as mean±SD, submitted to MANOVA and ANOVA, and compared using Bonferroni’s test. Morphology data (non- parametric variables) were expressed as median and interquartile range (IQR) to indicate central tendency and spread of data distribution respectively, submitted to Kruskal-Wallis test and between-group multiple comparisons were performed (Dunn’s test). Statistical significance was conventionally set at p≤0.05. SPSS^™^ 15.0 was used.

## Results

**Fig 1:**
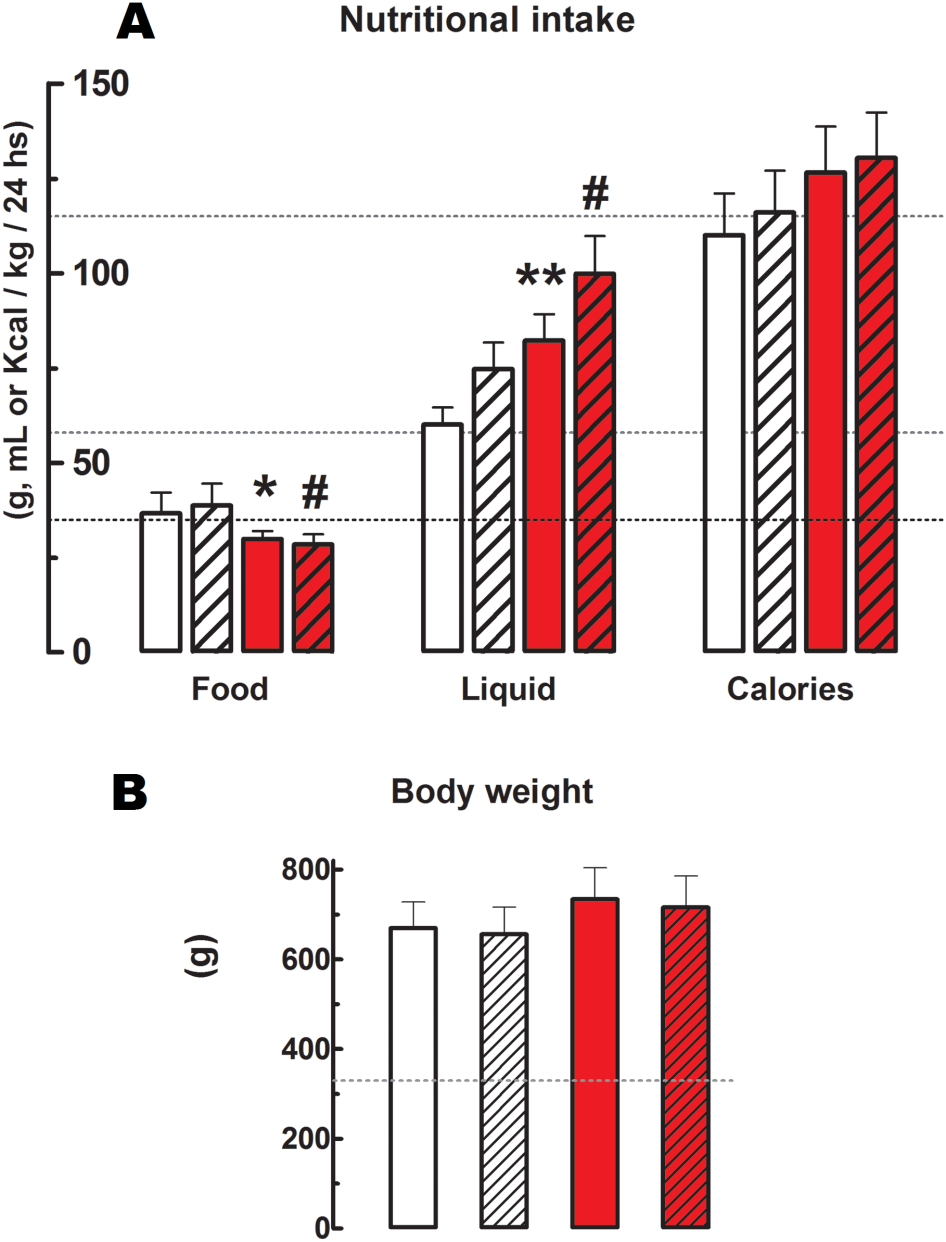
Nutritional data and body weight. A). Nutritional data in g, mL or Kcal/ kg body weight/ 24 hs. Calories calculation based on 3 Kcal/g of food and 0.42 Kcal/mL of cola drink. B). Body weight in g. Values are mean ± SD. Dotted lines indicate value at the beginning of the study. White bars: water sedentary, white hatched bars: water runner, red bars: cola sedentary, red hatched bars: cola runner. * p<0.05, ** p<0.01, *** p<0.001 vs water sedentary; **#** p<0.01 vs water runner.

**Fig 2:**
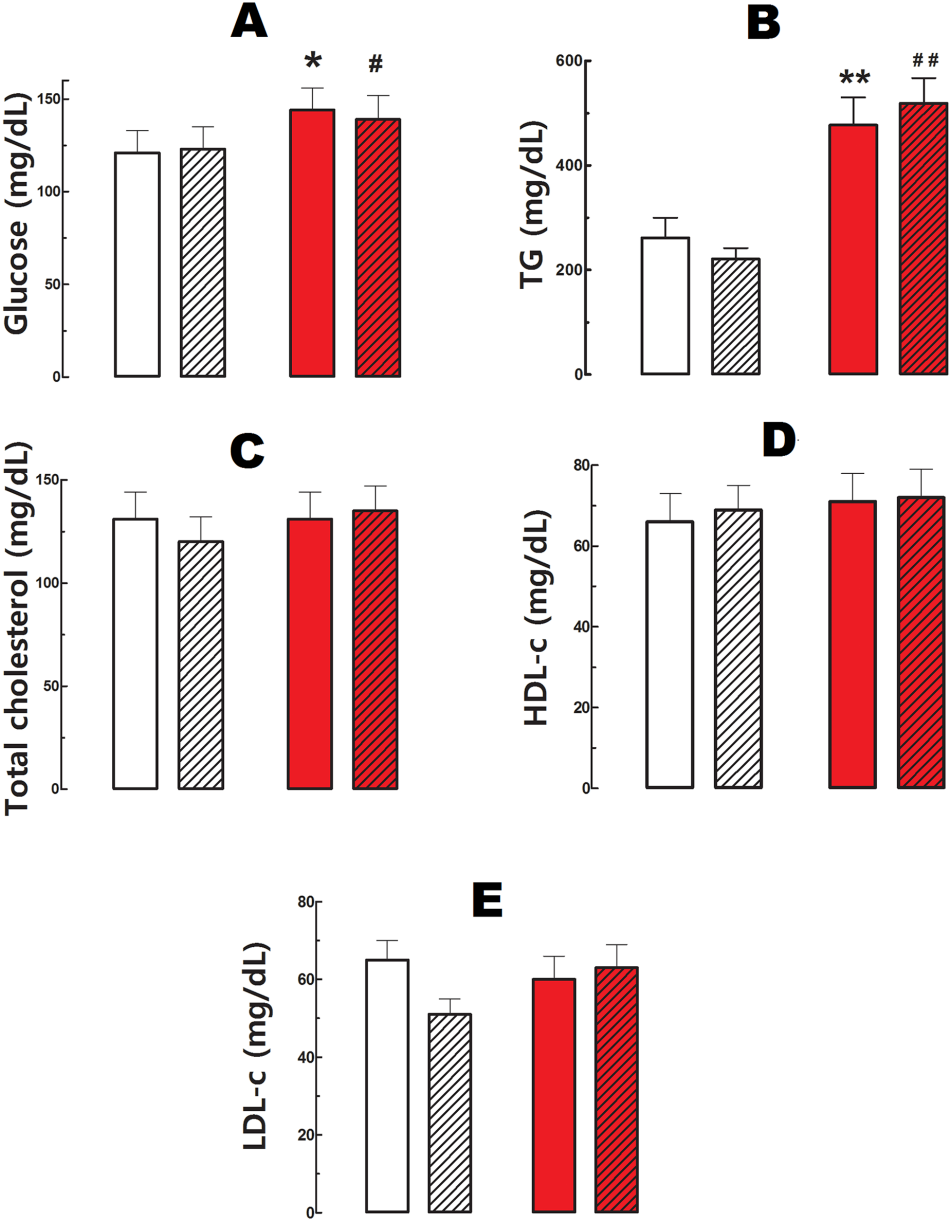
Plasma biochemical profile. A). Glucose in mg/dL. * p<0.05 vs water sedentary, # p<0.01 vs water runner. B). Triglycerides (TG) in mg/dL. ** p<0.01 vs water sedentary, ## p<0.01 vs water runner. C). Total cholesterol in mg/dL. D). High density lipoproteins (HDL) in mg/dL. E). Low density lipoproteins (LDL). Values are mean ± SD. White bars: water sedentary, white hatched bars: water runner, red bars: cola sedentary, red hatched bars: cola runner.

Cola-drinking (CS vs WS respectively) reduced food consumption -19% (p<0.05), increased liquid intake 37% (p<0.05) and calorie supply 15% (p<0.05) (Fig 1), and induced hyperglycemia (19%, p<0.05) and hypertriglyceridemia (82%, p<0.05) (Fig 2). Total cholesterolemia and SBP were not affected in CS compared with WS. Running did not modify nutritional data (Fig 1) or biochemical profile (Fig 2) either in cola-drinking rats (CR vs CS) or in water-drinking rats (WR vs WS). Overall, however, an increasing trend in liquid and calories intake was observed across groups: WS < WR < CS < CR (p<0.05).

Statistical analysis confirmed the existence of between-treatment interaction (p<0.001) for cola-drinking and running practice on pancreas morphology, masking their respective individual effects. Then, separate post-hoc analyses were performed in order to identify *per se* effects of each treatment.

**Fig 3:**
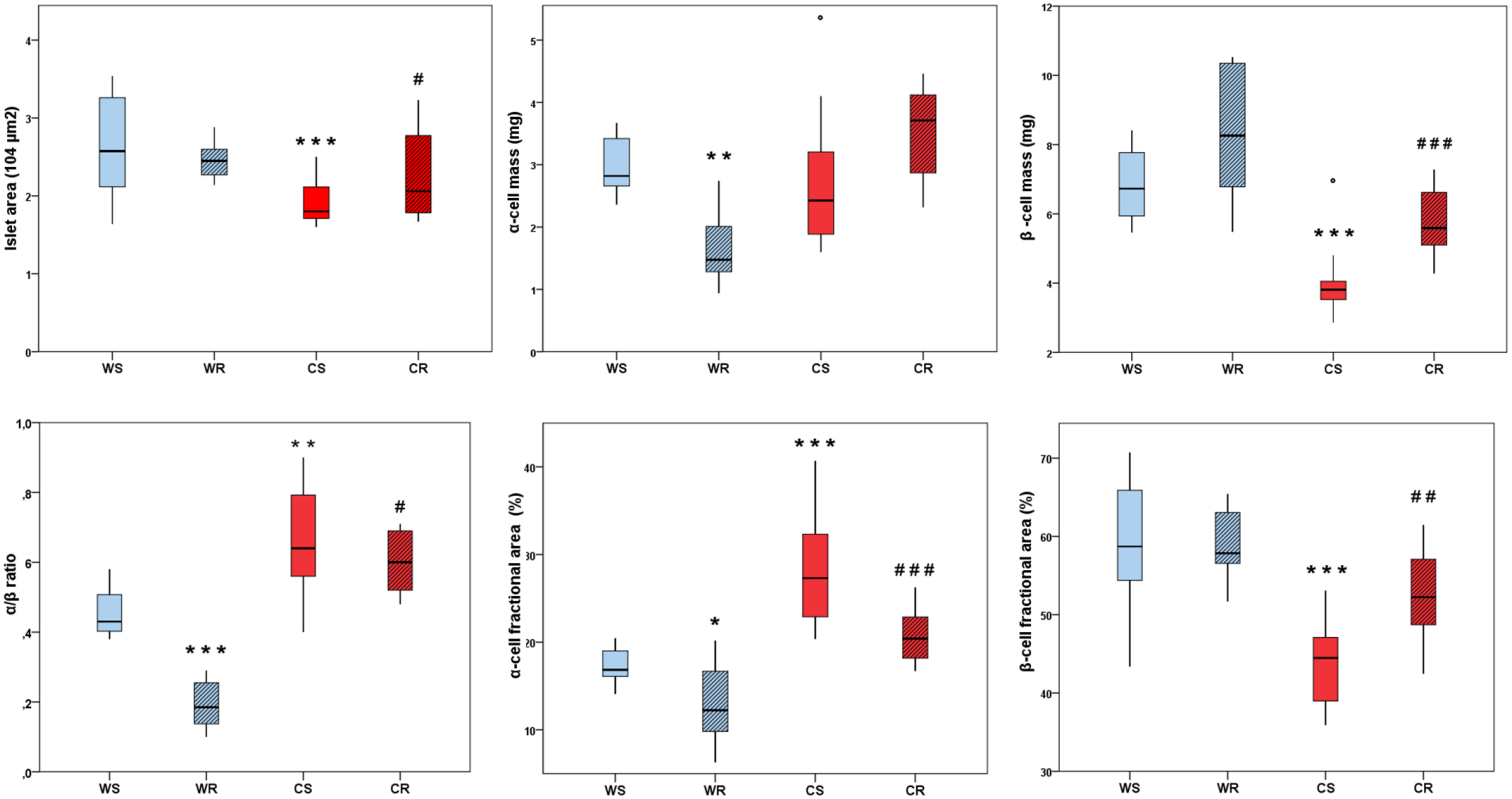
Quantitative morphology and immunohistochemistry of Langerhans islets. Boxplots show the median value (horizontal line), box limits indicate the 25^th^ and 75^th^ percentiles (box size covers the central 50% of the data), whiskers show extreme values of data distribution (maximal and minimal values within total range) and outliers are represented by **°**. * p<0.05, ** p<0.01, *** p<0.0001 vs WS; **#** p<0.05, ## p<0.001, ### p<0.0001 vs CS WS: water sedentary, WR: water runner, CS: cola sedentary, CR: cola runner. Values are expressed as mean ± SD. * p<0.05, ** p<0.01, *** p<0.001 vs WS; **#** p<0.01 vs WR.

Cola drinking (CS vs WS) reduced median pancreatic islet area by 30% (1.8 10^4^ μm^2^, IQR 1.71–2.11 vs 2.58 10^4^ μm^2^, IQR 2.12–3.26; p<0.0001), median β-cell mass by 43% (3.81 mg, IQR 3.52–4.05 vs 6.73, IQR 5.94–7.77; p<0.0001), median β-cell fractional area by 24% (14.24% units, 44.47, IQR 38.98–47.10 vs 58.71, IQR 54.59–65.89; p<0.0001), and increased median α-cell fractional area by 62% (+10.46% units, median 27.30, IQR 23–32 vs 16.84, IQR 16–19; p< 0.0001) and median α/β ratio by 49% (0.64, IQR 0.56–0.79 vs 0.43, IQR 0.40–0.51; p< 0.001) (Fig 3).

In water drinking rats (WR vs WS), running reduced median α-cell mass by 48% (1.48, IQR 1.28–2.01 vs 2.82, IQR 2.66–3.42; p <0.001), median α-cell fractional area by 27% (-4.61% units, 12.23, IQR 10–17 vs 16.84, IQR 16–19; p< 0.05) and median α/β ratio by 56% (-0.24% units, 0.19, IQR 0.14–0.26 vs 0.43, IQR 0.40–0.51; p<0.0001) (Fig 3).

Differently, in cola drinking rats (CR vs CS), running caused a 15% restoration of median islet area (2.06, IQR 1.78–2.78 vs 1.80, IQR 1.71–2.01; p<0.05), increased median β-cell mass by 47% (5.59, IQR 5.10–6.62 vs 3.81, IQR 3.52–4.05; p <0.0001) and median β-cell fractional area by 17% (7.77% units, 52.24, IQR 48.71–57.07, vs 44.47, IQR 38.98–47.10; p< 0.001), and reduced median α-cell fractional area by 25% (-6.89% units, 20.41, IQR 18–23 vs 27.30, IQR 23–32; p<0.0001) and median α/β ratio by 6% (-0.04% units, 0.60, IQR 0.52–0.69 vs 0.64, IQR 0.56–0.79; p<0.05) (Fig 3). Change in median islet area correlated significantly with change in median β-cell mass across groups (p<0.01).

**Fig 4:**
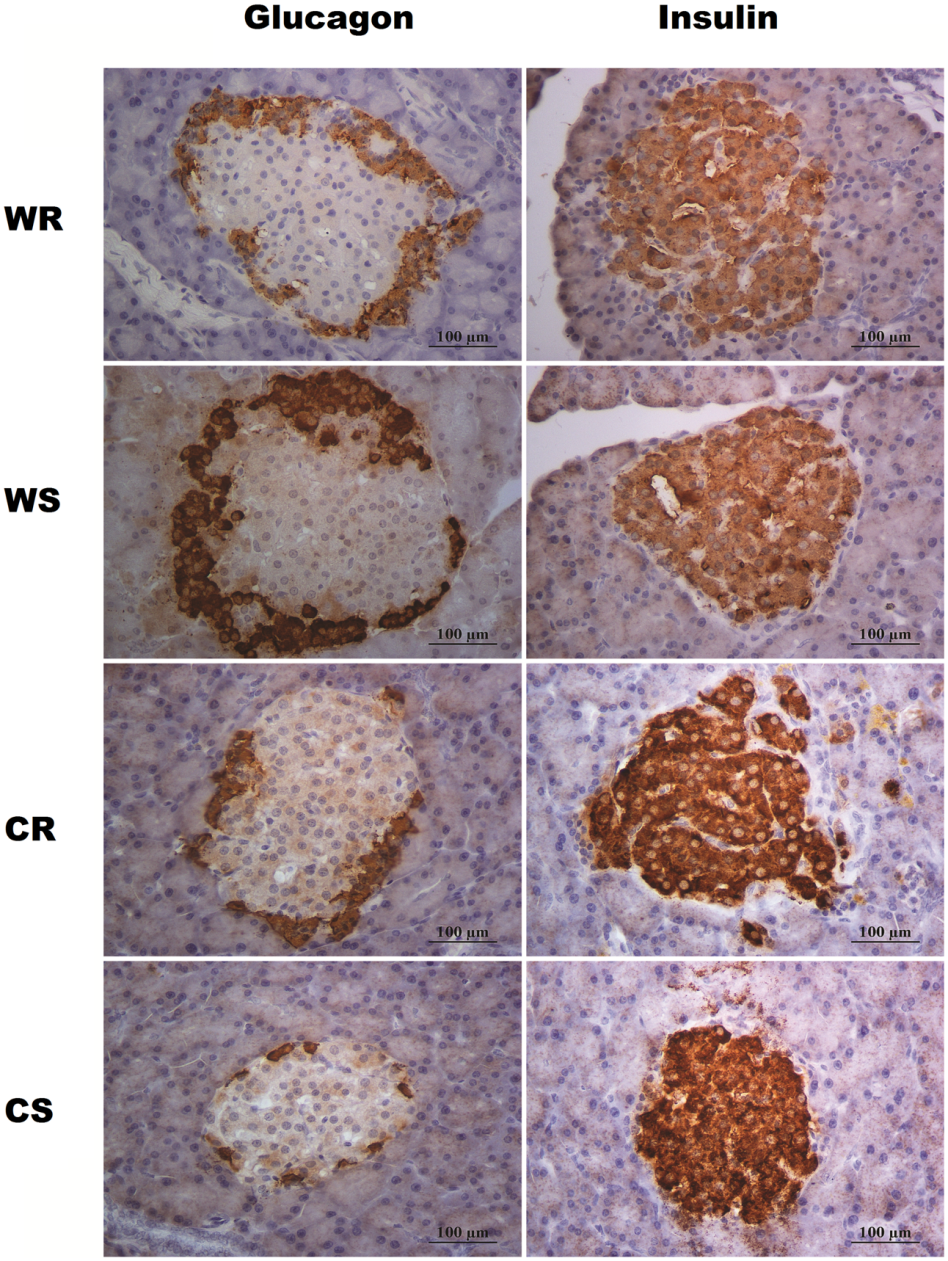
Immunolabeling for insulin and glucagon in Langerhans islets. Representative photomicrograph showing the effects of either chronic cola-drinking or running respectively or both as a combined treatment, on α- and β-cell area and islet size. Cytoplasmic expression of insulin (β-cells) and glucagon (α-cells) in all experimental groups, showing the usual rodent islet architecture. In WS and WR, the core of the islet is exclusively composed of insulin-immunopositive cells as expected, whereas glucagon immunopositivity is typically localized at the islet periphery. In CS and CR, glucagon-positive immunostaining is observed in the periphery, with focal extension into the center of the islet. Insulin-positive immunostaining is localized in the central portion of the islet, though leaving spots free of immunostaining with focal distribution.

Qualitative immunohistochemical findings are shown in Fig 4. Cola drinking reduced islet size and insulin immunopositive area (CS vs WS). Running *per se* (WR vs WS) showed no major changes on immunolabeling. However, in cola-drinking rats, running increased insulin immunopositive area and islet size (CR vs CS). In all cases, typical cells arrangement was found (Fig 4).

## Discussion

Cola drinking induced overall changes in plasma biochemistry and in pancreatic islets morphology in agreement with previous reports [22–24]. Previously, we reported replication of most features of metabolic syndrome in our experimental model of cola drink consumption. Body weight gain, hypertension, decreased food intake, hyperglycemia, hypertriglyceridemia, and a tendency to hypercholesterolemia were found after chronic consumption of sucrose-sweetened cola beverage in rats [22,23], along with insulin resistance, a substantial decrease in number of β cells (−58%) and increase in α/β-cell ratio [24].

In regard to the work by Otero-Losada et al [24], in both studies, 6 months cola-drinking reduced food consumption (-19% present, -31% previous), increased liquid intake (37% present, 69% previous) and calorie supply (15%, 12% previous) and induced hyperglycemia (19% present, 16% previous), hypertriglyceridemia (82% present, 2.8-fold previous) and did not affect total cholesterolemia. Body weight increased by 7% following 6 months cola drinking in both studies. Therefore, features of the experimental model were reproduced. We interpret differences in the extent of the observed changes between present and previous report, as a result of biological rhythms and seasonal variation.

Hypertriglyceridemia can be caused by fasting and lower food intake due to mobilisation of lipids from fat tissue. However, in this study and in our previous studies related to the effects of chronic cola beverage consumption, chronic cola drinking rats were not fasting, they actually drank plenty of cola beverage with large amounts of high fructose corn syrup therein. Actually, glycemia was higher than observed in their water drink counterparts. Cola drinking has been reported to increase adipocyte size [30]. On the other hand, considering that caffeine is a non-selective antagonist of A_1_ adenosine receptors and inhibits phosphodiesterase activity, lipolysis might be stimulated in cola drinking rats. The A_1_ adenosine receptors present on adipocytes are involved in the control of fatty acid uptake and lipolysis [31]. However, tracing the source of hypertriglyceridemia was not the aim of this study while it is indeed an interesting avenue to investigate in future studies in this line of research.

Present results are comparable to those reported previously [24] as far as changes are concerned, yet values are not the same from a quantitative point of view since different rats were studied.

Overall changes in pancreas morphology were similar in current and previous report [24]. However, different measures were obtained and other determinations were performed in respectively different experiments according to the aim of our previous work which far differs from the purpose and topic of the current study.

The relationship between exercise and food intake is complex and quite often inconsistent or conflicting results reflect this complexity. There exists a belief that physical activity drives up hunger while several independent variables affect the results of exercise-food intake relationship such as exercise protocol, individual characteristics, environmental conditions, seasonal period, and others [32,33]. In our experiment, increased energy intake should be expected in running groups. However, the idea that energy intake should increase by running may be arguable since, contrary to expectations, when forced to run on treadmills, energy intake of laboratory rats usually decreases in males [33]. In contrast, voluntary running (in running wheels) usually results in an increase in energy intake [33]. Conversely, energy intake usually increases in response to exercise in man [33]. Actually, increased energy intake is usually observed both in laboratory rats and in man, in response to cessation of exercise [33]. Besides, a moderate exercise protocol was used in this study in contrast with prolonged strenuous exercise which performed on a regular basis actually causes an increase food intake [34]. Currently, running practice failed to reduce hypertriglyceridemia in cola-drinking rats.

Interestingly, rather than simple summation, running and cola-drinking showed mutual interaction concerning with the effects on endocrine pancreas morphology. Running ameliorated some of the detrimental effects of cola-drinking on pancreatic islet morphology. The median islet area was significantly higher in CR compared with CS. Regardless the respective medians differed by 0.22 10^4^ μm^2^, islet area data was largely spread to higher values (positive skewness of the distribution) in CR compared with CS suggesting that individual differences might be actually larger for higher islet area values. From a physiological viewpoint, a far more important finding was the increase in β-cell population induced by running in cola-drinking rats. The increase of nearly 50% in β cells mass by running in cola drinking rats is by far the most relevant finding in this study.

Physiologically dynamic interaction between cola-drinking and running practice interaction, strongly supported by statistical interaction as well, might unfold in the critical contingency of systemic and local inflammatory and oxidative conditions associated with hypertriglyceridemia [35,36]. Hypertriglyceridemia is itself ruled out as the requirement for interaction since it was not affected in CR group, compared with CS. Sustained consumption of fructose, derived from chronic high fructose corn syrup in cola in this study, is known to lead to inflammation and reactive oxygen species production [37]. Actually, inflammation and oxidative environment secondary to hypertriglyceridemia may serve as ground for interaction between running and cola drinking treatments.

Differences in either behavioral traits or vegetative signs of catecholaminergic stimulation such as increased heart rate or blood pressure, were not observed in relation with drinking fluid in the present study. Particularly, no signs of psychomotor stimulation were observed in cola drinking rats in agreement with a previous report [38]. In that study, 2 month-old male rats were supplemented with caffeine 0.04% or 0.08% in the diet. The estimated caffeine intake was approximately 20 and 40 mg/kg per day respectively. At 90 days of life, the results indicated that intake of caffeine did not increase locomotor and exploratory activities [38].

It is unlikely that behavioral changes might have gone undetected in our study. Laboratory technicians and personnel staff responsible for animal handling and care, bear years-long practical experience in behavioral studies, have received specialized professional training and have developed a keen eye on behavioral traits. Besides, some of the authors of this study have published behavioral findings [39, 40].

Changes in β-cell mass were overall the highest contributors to the variation in islet area as confirmed by significant β-cell mass to islet area correlation across groups. This is inferable considering that the insulin-producing β-cells are the most abundant cell phenotype residing in the islets of Langerhans, and any change in β-cell mass is likely to yield a corresponding change in islet area.

Genetic deficiency of glucagon receptor prevents β-cell loss in experimental diabetes induced in glucagon knock-out mice [41]. Beta-cell loss, observed in CS group, was not found in CR group in our study. The role of α-cell in β-cell loss, the increase in α/β ratio and susceptibility to diabetes associated with long-term cola drinking, along with almost 50% increase in beta cells mass due to running in cola drinking rats, guarantees the continuation of our research.

Cola drinking for 6 months, induced insulin resistance, hyperglycemia and β-cell loss with increase in α-cell fractional area [24], affected kidney morphology [25], and did not affect rat behavior in the present study. Conversely, cola drinking for 3 months reduced blood glucose levels during an oral glucose tolerance test, suggesting improved insulin sensitivity and had no effect on kidney morphology [42], and increased locomotion [43]. Male Wistar rats, 2 month old were evaluated and ad libitum drinking was allowed in all four studies above mentioned and could not possibly account for discrepant findings. However, different local sugar-sweetened cola beverages were used. Celec et al used Kofola^™^ (sugar 80 g/L, caffeine 100 mg/L), Coca cola^™^ (sugar 110 g/L, caffeine 100 mg/L) and Pepsi cola^™^ (sugar 115 g/L, caffeine 110 mg/L) [42, 43]. In contrast, we use Coca cola^™^ in our studies. In Celec et al report, daily drinking volume consumed by rats in all cola groups was up to three times higher than the water intake in the control group [42]. In the present paper, drinking volume was 37% higher in cola drinking rats compared with control water drinking rats resulting in less caffeine consumption compared with Celec et al experiment [41]. However, we do not exclusively attribute discrepancies in behavioral changes to a lower caffeine dose.

Actually, the pivotal difference between our studies, including the present one, and Celec et al reports [41,43] appears to be the length of cola drinking period. We agree that, as stated by Celec et al’ s, despite 3 months of very high daily intake of cola, the duration of their study might have been too short. Time-dependency may not only account for their negative findings on renal morphology in contrast with epidemiological evidence of cola deleterious effects on kidney and with our own recent study [25], but also for the differences in both insulin sensitivity (increased at 3 months in reference 41 vs decrease at 6 months in reference 24] and behavior, between present and previous studies [42,43]. We agree that there may be multiple potential reasons for discrepancies as mentioned previously [41].

Contrasting with the effects of exercise on the cardiovascular system, experimental evidence of the effects of exercise training on pancreas is scarce and has been obtained in rats submitted to genetical, surgical or pharmacological manipulation, not to mention that α-cells were not evaluated in those protocols. Alternatively, we present a model combining a nutritional factor (cola drinking) with the practice of an exercise routine, a frequent combination in daily human life.

## Conclusions

This study is likely the first reporting experimental evidence of a beneficial effect of exercise on pancreatic morphology in cola-drinking rats by partially restoring β-cell population. An interesting physiological running-cola drinking interaction was observed. Moderate running advisably indicated in cola consumers and patients at risk of diabetes is supported by present findings.

Awareness that a healthy life style including the regular practice of moderate exercise and a balanced diet according to individual needs seems to be definitely the best prevention ever.
